# Influence of retinoblastoma-related gene silencing on the initiation of DNA replication by African cassava mosaic virus Rep in cells of mature leaves in *Nicotiana benthamiana *plants

**DOI:** 10.1186/1743-422X-8-561

**Published:** 2011-12-28

**Authors:** Gareth Bruce, Mei Gu, Nongnong Shi, Yule Liu, Yiguo Hong

**Affiliations:** 1 Research Centre for Plant RNA Signalling, College of Life and Environmental Sciences, Hangzhou Normal University, Hangzhou 310036, China; 2 Warwick HRI, University of Warwick, Wellesbourne, Warwick CV35 9EF, UK; 3 Biological Sciences Research Unit, University of Glamorgan, Pontypridd, CF37 1DL Wales, UK; 4 Clinical Sciences Research Institute, University of Warwick, Coventry CV2 2DX, UK; 5 School of Life Sciences, Tsinghua University, Beijing 100084, China

**Keywords:** *African cassava mosaic virus *(ACMV), DNA replication, Replication initiator protein (Rep), Retinoblastoma-related protein (RBR), Proliferating cell nuclear antigen (PCNA), Virus-induced RNA silencing (VIGS)

## Abstract

**Background:**

Geminiviruses mainly infect terminally differentiated tissues and cells in plants. They need to reprogramme host cellular machinery for DNA replication. This process is thought to be mediated by inactivation of cell-cycle repressor proteins and by induction of host DNA synthesis protein expression through actions of the geminviral replication initiator protein (Rep).

**Findings:**

Exploiting a *Nicotiana benthamiana *pOri2 line, which is transformed with a transgene consisting of a direct repeat of the African cassava mosaic virus (ACMV)-replication origin (Ori) flanking a non-viral DNA region, and virus-induced RNA silencing (VIGS), the impact of host gene expression on replication of the ACMV-derived replicon was investigated. The ACMV Rep trans-replicated the viral episomal replicon in leaves of young but not older pOri2 plants. Upon VIGS-mediated down-regulation of *N. benthamiana NbRBR1*, the retinoblastoma-related protein gene coding for a negative cell-cycle suppressor, recovered the ability of ACMV Rep for trans DNA replication, whereas the silencing of *NbPCNA *coding for the sliding clamp of DNA polymerase had no effect.

**Conclusions:**

These results suggest that the cellular machinery for DNA replication in differentiated tissues of older leaves cannot be reprogrammed by Rep alone but may need other uncharacterised viral and plant factors.

## Introduction

African cassava mosaic virus (ACMV) is a single-stranded (ss) DNA virus in the genus *Begomovirus*, family *Geminiviridae*. ACMV possesses two circular DNAs, designated DNA-A and DNA-B of approximately 2.7 kb [1], both are required for systemic infection of plants [2]. DNA-A and DNA-B share an almost identical common region that contains *cis*-acting elements required for replication and transcriptional modulation of viral gene expression [3,4]. The bipartite ACMV genome encodes eight proteins that are responsible for the viral life cycle in and among host plants. The multifunctional replication initiator protein (Rep) is essential for the initiation of rolling circle replication (RCR) of both DNA A and DNA B [5,6]. Rep also acts as a transcription repressor [7] and can trigger hypersensitive response and viral resistance in plants [5,6,8,9]. ACMV infection can induce antiviral RNA silencing defence [10], affect siRNA production [11], disturb microRNA biosynthesis and cause abnormal developmental phenotypes in plants [12,13].

## Results and discussion

**Figure 1:**
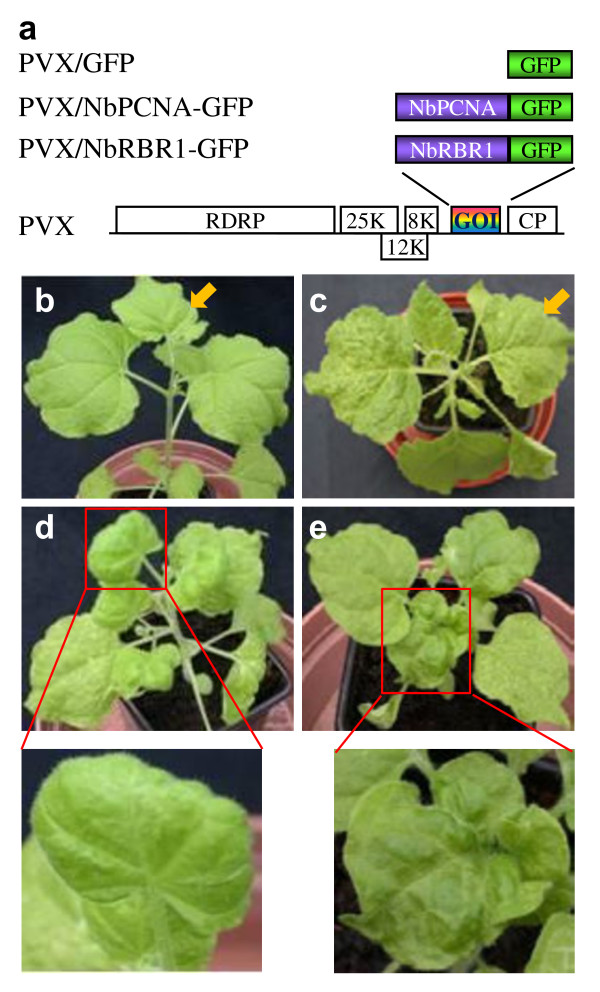
**Virus induced RNA silencing (VIGS) (a) Construction of PVX/NbRBR1-GFP and PVX/NbPCNA-GFP in PVX/GFP**. The PVX genome organisation is shown to encode the RNA-dependent RNA polymerase (RDRP), the triple-gene block (25 K, 12 K and 8 K) and the capsid protein (CP) as well as an insertion of gene of interest (GOI). (b-e) VIGS phenotypes of transgenic *Nicotiana benthamiana *line pOri2 plants. Plants (25 days old) were mock-inoculated (b) or inoculated with PVX/GFP (c), PVX/NbRBR1-GFP (d) or PVX/NbPCNA-GFP (e). PVX/GFP infected plant showed mosaic and chlorosis at 10 days post-inoculation (dpi) whilst mock inoculated plant remained healthy. Plants challenged with either PVX/NbRBR1-GFP or PVX/NbPCNA-GFP showed local and systemic infection at early stage (7-14 dpi) of viral invasion, but later on (approximately 21 dpi onwards) newly growing leaves showed no viral symptoms. VIGS of *NbRBR1 *or *NbPCNA *caused abnormal leaf development and growth retardation. Plants (50 days old) were photographed at 25 dpi. The boxed portions of plants are enlarged to show VIGS-related phenotypes. Leaves of plants with mock inoculation (b) or infected with PVX/GFP (c) at the same developmental stage as these boxed leaves are indicated with an arrow. There leaves were infiltrated with agrobacterium and subsequently used for sqRT-PCR and PCR (see Figures 2 and 3).

An *in planta *trans-replication system [5] was utilised to investigate the impact of *Nicotiana benthamiana *retinoblastoma-related (NbRBR1) and proliferating cell nuclear antigen (NbPCNA) genes, (which code for a repressor of cell cycle and the sliding clamp of host DNA replicase, respectively), on viral DNA replication. The potato virus X (PVX)-based vector for virus induced RNA silencing (VIGS) was employed to knock-down *NbRBR1 *and *NbPCNA *expression. To achieve this, a 420-bp fragment corresponding to the 5' end of *NbRBR1 *(Genbank accession number: AY699399) was obtained through reverse-transcription (RT)-PCR with total RNAs template from healthy *N. benthamiana *leaves using Qiagen RNeasy Minikit (Qiagen) for extraction and the primer pair PP369 (5' ACGACATCGATATGGGTGGAGCTGAATAATTGTTC 3') and PP449 (5' CTCTTCCGGCCGTCTGAACCATACAGATTG 3'). The RT-PCR product was digested with *Cla*I/*Eag*I and cloned into the same sites of the modified PVX/GFP vector to produce PVX/NbRBR1-GFP (Figure 1a). Similarly, the 5'-end 371-bp fragment of the *N. benthamiana NbPCNA *(Genbank accession number: AF303075) was obtained through RT-PCR using a pair of primers PP398 (5' TTTTCAATCGATATGTTGGGAATTACGGCTTGT 3') and PP450 (5' AATTACCGGCCGGTCACCGATTACTGCTAAGGT 3') to generate PVX/NbPCNA-GFP (Figure 1a). Correct insertions were confirmed by sequencing.

In each of three separate experiments, 3-4 young transgenic *N. benthamiana *line pOri-2 plants (25 days old) were inoculated with RNA transcripts for PVX/GFP, PVX/NbRBR1-GFP or PVX/NbPCNA-GFP as previously described [9]. The transgenic pOri2 line contains a transgene consisting of a direct repeat of the ACMV replication origin (Ori) flanking a non-viral DNA region as previously described [5,6]. In contrast to mock-treated controls [plants were inoculated with RNase-free water] (Figure 1b), plants inoculated with recombinant PVX RNAs developed local chlorotic lesions at 4-6 days post-inoculation (dpi). Subsequently, mosaic and chlorosis appeared in the systemic young leaves at approximately 10 dpi, which were maintained in plants infected with PVX/GFP (Figure 1c). By contrast, plants systemically infected with PVX/NbRBR1-GFP or PVX/NbPCNA-GFP started to show recovery from viral infection at approximately 14 dpi and typical PVX symptoms disappeared almost completely at 25 dpi. In contrast, VIGS of *NbRBR1 *resulted in growth retardation, abnormal leaf development, and newly emerged leaves were irregularly shaped and had a definite curl downwards running the whole circumference of the leaf (Figure 1d). VIGS of *NbPCNA *also caused stunted growth and distorted leaves. Young leaves growing at the apical meristem were heavily crinkled, curled upwards and rosette in shape (Figure 1e). These phenotypic changes are similar to that previously described for suppression of *NbRBR1 *or *NbPCNA *in *N. benthamiana *using various VIGS systems [14,15].

**Figure 2:**
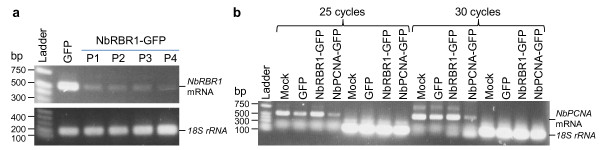
**VIGS reduction of *NbRBR1 *and *NbPCNA *expression (a) Semi-quantitative reverse transcription PCR (sqRT-PCR) assays of *NbRBR1 *mRNA levels in transgenic *Nicotinana benthamiana *line pOri-2 plants inoculated with PVX/GFP (GFP) or PVX/NbRBR1-GFP (NbRBR1-GFP, P1-P4 for four individual plants)**. All plants were at the age of 56 days. (b) SqRT-PCR assays of *NbPCNA *mRNA levels in healthy pOri2 plant (mock), or plants inoculated with PVX/GFP (GFP), PVX/NbRRB1-GFP (NbRBR1-GFP), or PVX/NbPCNA-GFP (NbPCNA-GFP). SqRT-PCR of 18S rRNA indicates similar amount of total RNAs used in each sample. The positions and sizes of 1-kb DNA marker (Ladder) and positions of RT-PCR products specific for *NbRBR1 *and *NbPCNA *mRNA as well as 18S rRNA are indicated.

VIGS of *NbRBR1 *or *NbPCNA *in pOri2 plants was further confirmed by semi-quantitative (sq) RT-PCR. Total RNAs (100 ng) were extracted at 31 dpi from leaves at the age of 56-days old plants, and used as templates together with one of the two sets of primers PP369 and PP370 (5' TCTTGCGGCCGTCGCTTGTAGTACTTGCTTAAAAG 3') or PP398 and PP439 (5' TTGAGACGGCCGCACCTTCCTTTGTCGCAGAAATTA 3') for sqRT-PCR assays. These primers allowed a specific detection of the 500-nt portion corresponding to the endogenous mRNA transcripts of the two target genes. Reduction in *NbRBR1 *(Figure 2a) or *NbPCNA *(Figure 2b) expression was obvious in the plants inoculated with PVX/NbRBR1-GFP or PVX/NbPCNA-GFP, respectively, when compared to that in mock-inoculated plants or plants infected with PVX/GFP. It was also noted that silencing of *NbPCNA *did not affect the level of *NbRBR1 *mRNA (Figure 2b). The equal amount of total RNAs in each of the sqRT-PCR reactions was verified by detecting a similar level of 18S rRNA in all samples using primers PP271 (5'CGGCTACCACATCCAAGGAAGG 3') and PP272 (5' GAGCTGGAATTACCGCGGCTG 3') (Figure 2a, b). Taken together, these results demonstrate that VIGS of endogenous *NbRBR1 *and *NbPCNA *expression occurred and the aberrant phenotypes observed were caused by silencing of *NbRBR1 *or *NbPCNA*.

**Figure 3:**
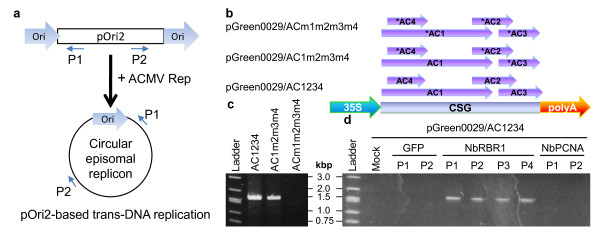
**VIGS of *NbRBR1 *and *NbPCNA *affects episiomal DNA replication (a) Outline of the trans-DNA replication system in the transgenic pOri2 line **[5,6] . The replication origin (Ori) of the African cassava mosaic virus (ACMV) and the P1/P2 primers for PCR detection of the circular episomal replicon in the presence of ACMV Rep are indicated. (b) Constructs for pGreen0029/AC1234, pGreen0029/AC1m2m3m4 and pGreen0029/ACm1m2m3m4 expressing the ACMV complementary-sense genes (CSG, AC1 - AC4], AC1 alone or no gene under the control of the CaMV 35S promoter and polyA signal. Mutated genes are indicated by asterisks. (c) PCR assays for circular episomal replica in young *N. benthamiana *line pOri-2 plants at the age of 31 days. In three separate experiments, young growing leaves were infiltrated with *Agrobacteria *carrying pGreen0029/AC1234 (AC1234), pGreen0029/AC1m2m3m4 (AC1m2m3m4), or pGreen0029/ACm1m2m3m4 (ACm1m2m3m4). (d) Impact of VIGS-mediated reduction of *NbRBR1 *or *NbPCNA *expression on viral replication. In each of three experiments, line pOri2 plants (25 days old) were mock-inoculated (Mock), or inoculated with PVX/GFP (GFP, two plants P1-P2), PVX/NbRBR1-GFP (NbRBR1, four plants P1-P4) or PVX/NbPCNA-GFP (NbPCNA, two plants P1-P2). At 25 days post-inoculation, leaves of these plants (50 days old) as indicated in Figure 1 were subsequently infiltrated with *Agrabacteria *carrying pGreen0029/AC1234 and analysed by sqRT-PCR (see Figure 2) and PCR 6 days later. The positions and sizes of 1-kb DNA marker (Ladder) are indicated.

The effect of silencing of *NbRBR1 *or *NbPCNA *on the life cycle of ACMV, particularly in terms of DNA replication, was investigated in the pOri2-based trans-DNA replication system (Figure 3a). Trans-DNA replication to generate a circular episomal replicon occurs in the presence of ACMV Rep [5,6]. Mature leaves of healthy and PVX/GFP-infected pOri-2 plants, and leaves of pOri-2 plants which recovered from viral infection and showed phenotypic responses to *NbRRB1 *or *NbPCNA *silencing were infiltrated with *Agrobacterium tumefaciens *LBA4404 carrying pGreen0029/AC1234, pGreen0029/AC1m2m3m4 or pGreen0029/ACm1m2m3m4 (Figure 3b). Expression ACMV complementary strand genes (CSG) including Rep (AC1) from these cassettes is under the control of the 35S promoter and the CaMV polyadenylation signal. An equivalent amount of ACMV CSG RNA transcripts transcribed from/AC1234, pGreen0029/AC1m2m3m4 or pGreen0029/ACm1m2m3m4 was detected in agro-infiltrated leaf tissues by sqRT-PCR (unpublished data). Episomal circular DNA was readily detectable by PCR using primers P1 (5' TCGCGCTGATACCAGACGTTGC 3') and P2 (5' GGACTGGCATGAACTTCGGTG 3') (Figure 3a) and template DNA (100 ng) extracted from infiltrated leaf tissues at 6 days post agro-infiltration. Expression of Rep alone (pGreen0029/AC1m2m3m4) or together with AC2, AC3 and AC4 (pGreen0029/AC1234) in growing leaves of young healthy pOri2 plants at the age of 31 days, was able to initiate trans-replication. PCR (Figure 3c) detected a specific 1.6-kb fragment derived from episomal replica whilst no such DNA was detected in leaves infiltrated with pGreen0029/ACm1m2m3m4 carrying agrobacteria as negative control, from which no ACMV gene product is translatable. At the older age (56 days), agro-infiltrated leaves of mock-, PVX/GFP, or PVX/NbPCNA-infected pOri2 plants were unable to trans-replicate the episome (Figure 3a). However, if *NbRBR1 *expression was reduced by VIGS (Figure 2a), circular episomes were readily detected (Figure 3d). Similar results were obtained in *NbRBR1*-silenced old leaves if Rep was expressed alone from pGreen0029/AC1m2m3m4 (data not shown). These data suggest that the expressed ACMV Rep is only able to initiate DNA replication in older leaves if expression of the cell cycle block NbRBR1 is reduced.

Geminiviral Rep is not a DNA polymerase. However, during viral DNA replication the oligomeric Rep protein cleaves the viral replication origin TAATATT↓AC and acts as an ATP-dependent ligase to re-circularise progeny ssDNA [3,16,17]. Efficient viral DNA replication is also dependent on functional interplays between Rep and other viral proteins including the replication-enhancing protein and coat protein [18,19], as well as with host factors such as replication factor C [20,21]. Moreover, geminiviruses infect terminally differentiated tissues/cells in which host DNA polymerases are not functional. Therefore, to establish infection, geminiviruses need to re-programme host cellular machinery for DNA replication. This process is thought to be mediated by inactivation of the cell-cycle repressor RBR protein through direct Rep-RBR interactions and by Rep-triggered induction of host DNA synthesis PCNA expression [22-25]. It is demonstrated that with the begomovirus *Tomato golden mosaic virus*, Rep-RBR interaction and PCNA accumulation are important for virus replication and infectivity in *N*. *benthamiana *[22,24,26]. On the other hand, in mastreviruses, such as Maize streak virus (MSV), an intact Rep RBR-interaction motif is not required for virus replication in culture cells or infectivity in maize, although it is possibly required for wild-type symptom development [27-29]. Indeed, wild-type MSV invades both vasculature and mesophyll cells of mature maize leaves. In contrast, MSV with a dysfunctional Rep RBR-interaction motif was restricted to the vasculature, in which dividing cells possess the active machinery for DNA synthesis. It is suggested that mature leaves contain high levels of RBR and the MSV Rep-RBR interaction is essential only in tissues with high levels of active RBR [28]. However, prior to Rep-mediated deregulation of cell-cycle control to provide an environment that is able to accommodate replication, the Rep gene must be transcribed from double-stranded (ds) DNA intermediates to express mRNA from which the Rep protein can then be translated. It remains an open question how a geminivirus generates dsDNA from its ssDNA genomes in cells where the replication machinery is inactive.

ACMV Rep was unable to initiate RCR in cells of mature leaves of older pOri2 plants although it was effective in triggering RCR in cells of growing leaves of young plants. It is possible that young growing leaves possess many S-phase cells with an active DNA replication machinery, which ceases to function in cells of older mature leaves. One factor affecting the cellular DNA replication capacity can be different expression levels of genes that encode cell cycle modulators such as *RBR1*. For instance, there may be low expression of *NbRBR1 *in S-phase cells of growing leaves of young plants whereas in older leaves high *NbRBR1 *expression could effectively maintain cells in G1 phase with minimal replication activity. Indeed, in older tissues *NbRBR1 *mRNA was readily detectable (Figure 2). The ACMV Rep protein can hijack a functional replication apparatus in S-phase cells of young leaves to instigate and trans-replicate the episome (Figure 3a). However, Rep alone, or together with other ACMV CSG products AC2, AC3 and AC4, cannot re-programme a "ceased" replication machinery through its interaction with RBR. On the other hand, it is not surprising that silencing of *NbPCNA*, an auxiliary protein of DNA polymerase, could not restore the deficiency of DNA replication (Figure 3d). However, reduction of *NbRBR1 *expression by VIGS may allow G1 cells of older leaves progressing into the S phase. Consequently, DNA synthesis functionality is re-activated and then exploited by ACMV Rep (Figure 3c-d). Thus, our findings suggest that some uncharacterised viral and/or plant factors may participate in reactivating the host cellular machinery for geminiviral DNA replication in terminally differentiated cells, and this process is a more complex one than previously proposed [22-25,30]. This idea is supported by the facts that the curtovirus C4 protein can induce plant cell cycle regulator gene expression [31] and that in fission yeast the ACMV Rep can affect cell division cycle despite no RBR homologue has been identified to date [32].

## Competing interests

The authors declare that they have no competing interests.

## Authors' contributions

GB designed and performed experiments; MG, NS and YL contributed through discussion and revised paper. YH initiated the project, designed experiments and wrote paper. All authors read and approved the final manuscript.
